# Sex-specific dynamics of MASLD reveal early hepatic and extrahepatic metabolic deterioration in females despite long-term protection

**DOI:** 10.1186/s13293-026-00876-y

**Published:** 2026-03-22

**Authors:** Maria Repollés-de-Dalmau, Anna Marsal-Beltran, Catalina Núñez-Roa, Joan Vendrell, Victòria Ceperuelo-Mallafré, Sonia Fernández-Veledo

**Affiliations:** 1 DIAMET Research Group, Institut de Recerca Biomèdica Catalunya Sud (Formerly Institut d’Investigació Sanitària Pere Virgili), Hospital Universitari Joan XXIII, Tarragona, 43005 Spain; 2 CIBER de Diabetes y Enfermedades Metabólicas Asociadas (CIBERDEM), Instituto de Salud Carlos III, Madrid, 28029 Spain; 3 Rovira i Virgili University, Reus, 43201 Spain

**Keywords:** Sexual dimorphism, Steatohepatitis, Gut, CDAHFD

## Abstract

**Background:**

Metabolic dysfunction-associated steatotic liver disease (MASLD) is a chronic condition characterized by hepatic fat accumulation and systemic metabolic dysfunction. MASLD exhibits clear sex differences, yet the mechanisms underlying these disparities remain poorly defined.

**Methods:**

To investigate the early temporal dynamics of MASLD, male and female mice were fed a choline-deficient, methionine-restricted (0.1%) high-fat (61%) diet (CDAHFD; characterized by impaired hepatic lipid export and enhanced lipotoxic stress) for two weeks, enabling assessment of initial metabolic responses.

**Results:**

Despite their presumed protection, females developed an exacerbated hepatic phenotype accompanied by intestinal remodeling and compromised barrier integrity. In contrast, males displayed early weight loss and improved glucose tolerance, alongside reduced hepatic transcriptional changes indicative of adaptive metabolism. Additionally, sex-specific hypothalamic responses were observed, with males showing reduced expression of microglial homeostatic markers, increased inflammation, and alterations in energy balance-related signaling, consistent with neuroimmune modulation. These responses were paralleled by sex-dependent alterations in adipose tissue, including early adipocyte remodeling and distinct transcriptional changes, which were predominantly consistent with baseline sex differences and therefore suggest a limited contribution of this tissue to the early diet-induced metabolic divergence. Importantly, these early sex-dependent adaptations were not sustained over time, as prolonged CDAHFD (14 weeks) resulted in a shift towards a more sever inflammatory and fibrotic hepatic phenotype in males, while females exhibited relative preservation of hepatic metabolic function and attenuation of intestinal alterations.

**Conclusions:**

These findings demonstrate that sexual dimorphism in MASLD arises early and evolves dynamically, with females mounting an acute metabolic and intestinal stress response, whereas males activate compensatory pathways that preserve metabolic homeostasis. These results highlight sex-specific trajectory in MASLD progression and emphasize the need for integrative approaches to unravel its pathophysiology.

**Supplementary Information:**

The online version contains supplementary material available at 10.1186/s13293-026-00876-y.

## Highlights

Females develop more early severe liver damage, accompanied by pronounced intestinal alterations and impaired gut barrier integrity under a choline-deficient, methionine-restricted high-fat diet (CDAHFD).

Males show early body weight loss, improved glucose tolerance, and reduced liver gene expression modifications in response to CDAHFD.

CDAHFD elicits sex-specific central and peripheral adaptations, including altered hypothalamic neuroimmune signaling and adipose tissue transcriptional remodeling.

Findings reveal that sex differences in MASLD appear early and progress along distinct biological pathways.

**Supplementary Information:**

The online version contains supplementary material available at 10.1186/s13293-026-00876-y.

## Abstract (plain language summary)

Metabolic dysfunction-associated steatotic liver disease (MASLD) is a common liver disorder where fat builds up in the liver and disrupts overall metabolism. It is known that men and women experience this disease differently, but the reasons for these differences are still unclear. To explore this, researchers studied male and female mice that were fed a special diet designed to cause early liver stress. Surprisingly, the female mice showed more severe liver changes than the males, even though females are often thought to be protected against such damage. The females also showed signs of intestinal changes and a weakened gut barrier, suggesting their bodies were under higher stress.

In contrast, male mice lost weight, had better blood sugar control, and showed reduced changes in the liver, which suggested that their bodies were adapting to the diet rather than being harmed by it. These differences were also linked to changes in the brain areas that control appetite, energy use, and inflammation. Overall, this study shows that sex differences in MASLD appear very early and follow different paths. Females respond with stronger stress and intestinal reactions, while males seem to trigger protective mechanisms. Understanding these early sex-specific patterns could help guide the development of better treatments for both men and women with liver disease.

**Supplementary Information:**

The online version contains supplementary material available at 10.1186/s13293-026-00876-y.

## Background

Metabolic dysfunction-associated steatotic liver disease (MASLD) is a chronic liver disorder characterized by pathological hepatic lipid accumulation and the presence of at least one cardiometabolic risk factor [1, 2]. Emerging evidence highlights a pivotal role of the gut microbiota in MASLD pathogenesis, where dysbiosis compromises intestinal barrier integrity, facilitating the translocation of bacterial products that amplify hepatic inflammation and metabolic derangements [3].

The global prevalence of MASLD has risen sharply, currently affecting approximately 38% of the adult population worldwide, equivalent to nearly two in five individuals, and representing a 50% rise over the past three decades [4, 5]. This escalating trend underscores a major public health challenge, with projections indicating further substantial increases in the coming years [6]. Prevalence is consistently higher in men than in women worldwide, and men exhibit a greater propensity for progression to metabolic dysfunction-associated steatohepatitis (MASH), fibrosis, and liver complications [4, 5]. Notably, sex-specific prevalence patterns vary across the lifespan as men experience peak MASLD incidence in early to mid-adulthood, followed by a decline after 50–60 years [7], whereas women show increasing prevalence after menopause, peaking between 60 and 69 years of age [8]. These differences are highly attributed to sex-dependent mechanisms that influence lipid metabolism, inflammation, oxidative stress and liver regeneration, with sex hormones among the key modulators [8]. Despite extensive clinical observations, the mechanistic basis driving these sex differences and their influence on metabolic progression remains poorly understood.

Preclinical models broadly reflect the MASLD pathophysiological features observed in humans [9–12] and reveal clear sex-related disparities, with females typically showing delayed or attenuated disease progression. However, most of the available evidence derives from mid- to long-term interventions, leaving the early molecular and cellular events that initiate the disease, and the potential sex-specific responses during these initial stages, largely undefined. Furthermore, the lack of consensus regarding the optimal mouse strain, diet composition, and feeding duration, together with the absence of systematic comparisons of disease progression between sexes, continues to hinder the elucidation of these mechanisms.

To address these gaps, we designed an experiment using a choline-deficient, methionine-reduced high fat diet (CDAHFD) in both male and female mice, focusing primarily on short-term (2 weeks) dietary responses, with complementary long-term (14 weeks) analysis. This model rapidly induces fibrosis while preserving body weight and reproducing key features of human MASH. Importantly, the inclusion of an early time point allowed us to capture initial molecular and metabolic responses before the onset of advanced pathological remodeling. By minimizing confounding effects from downstream consequences (e.g. fibrosis progression, tissue remodeling, microbiome drift), this strategy enables discrimination between primary sex-specific triggers of disease susceptibility and secondary adaptations arising during chronic liver injury.

Our findings demonstrate that sexual dimorphism in MASLD is dynamic and evolves over time. In our short-term dietary model, despite their presumed protection, females exhibit an early and pronounced hepatic response accompanied by structural alterations in the colon, while males show improved glucose tolerance and body weight loss under the same conditions. Notably, early CDAHFD exposure is associated with sex-divergent hypothalamic responses, characterized by enhanced pro-inflammatory signaling and alterations in microglial homeostatic markers expression in males, pointing to a distinct neuroimmune adaptations that may differentially shape systemic metabolic regulation. Collectively, these observations underscore the relevance of temporal dynamics in shaping sexual dimorphism during MASLD development and suggests that early female-specific alterations may precede, and potentially shape, the emergence of the protected phenotype typically observed at later stages.

## Methods

### Mice and diets

#### General

Animals were maintained at the Faculty of Medicine and Health Science animal facility of Rovira i Virgili University and were housed 6 per cage (for the 2-week experiment) and 3–4 per cage (for the 14-week experiment) under controlled conditions of 12-h light/dark at 22 °C with ad libitum access to food and water unless otherwise stated. Body weight and food intake were recorded weekly. Food intake was calculated by measuring total food consumption per cage, dividing this value by the number of mice housed per cage and days, and subsequently normalizing individual intake to body weight to obtain relative daily food intake per mouse. All animal studies were supervised and approved by the Rovira i Virgili University Animal Welfare and Governmental Ethics Committee (reference 10970). All experimental procedures involving animals conformed to the European Union Directive 2010/63/EU and the European Commission Recommendation 2007/526/EC on the protection of animals used for experimental and other scientific purposes, enacted under the Spanish Royal Decrees 53/2013 and 118/2021of the Spanish Ministry of Economy and Competitiveness.

#### 2-weeks experiment

9-week-old male and female wild-type (WT) mice on a C57BL/6 J background were purchased from Charles River Laboratories. Following a one-week acclimatation period, mice were assigned either a standard chow diet (CHD, n = 6 per sex) (72.4 kcal% carbohydrates, 8.4 kcal% fat, 19.3 kcal% protein, 2800 mg methionine, 1600 mg choline; A04, Safe Diets), or L-Amino Acid Rodent Diet With 0.1% Methionine and No Choline (CDAHFD, n = 6 per sex) (21 kcal% carbohydrates, 61 kcal% fat, 18 kcal% protein, 800 mg methionine, 0 mg choline; A06071302, Research Diets) with *ad libitum* access to food and water. One week and a half later, metabolic tests were conducted. Two weeks after the start of the diet, and after an overnight fasting, mice were sacrificed by cardiac puncture under isoflurane anesthesia. Blood, liver, adipose tissues, hypothalamus, and colon were collected for further analysis (mRNA and protein expression and histological analyses). Animals selected for histological analyses were chosen based on body weight prior to sacrifice, selecting those mice closest to the group’s mean.

#### 14-weeks experiment

10-week-old male and female WT mice on a C57BL/6 J background were fed either a CHD (n = 3) or a CDAHFD (n = 4) for 14 weeks. Metabolic tests were performed prior to diet initiation and during the week before the sacrifice. At the end of the feeding period, mice were fasted for 2 h and culled by cardiac puncture under isoflurane anesthesia. Liver tissue, hypothalamus, and colon were collected for further analysis (mRNA expression and histological analyses). Animals selected for histological analyses were chosen based on body weight prior to sacrifice, selecting those mice closest to the group’s mean.

### Glucose tolerance test (GTT)

Glucose (2 g/Kg BW) was intraperitoneally administered after a 6-h fast, and blood glucose levels were monitored with a glucometer and glucose strips at baseline (time 0) and at 15, 30, 60, 90 and 120 min post-injection. Blood samples collected at baseline and 15 min after glucose administration were centrifuged at 2000 g for 5 min at 4ºC to obtain plasma. Plasma insulin levels at both time points were subsequently quantified using a Mouse Insulin ELISA kit (10-1247-01, Mercordia), according to the manufacturer’s instructions.

### Insulin tolerance test (ITT)

After a 3-h fast, male and female mice received an intraperitoneal injection of human insulin (Actrapid, Novo Nordisk) at a dose of 0.75 U/Kg of BW. Blood glucose levels were determined prior to the injection (0 min) and at 15, 30, 60, 90, and 120 min post-injection.

### Pyruvate tolerance test (PTT)

For the pyruvate tolerance test (PTT), mice were fasted for 16 h before receiving an intraperitoneal injection of sodium pyruvate (P2256, Sigma Aldrich) (1.5 g sodium pyruvate/kg). Glucose levels were determined before the injection and at 15, 30, 60, 90, and 120 min after.

All metabolic tolerance tests (GTT, ITT, PTT) were performed sequentially in the same cohort of mice, with a minimum washout period of 3 days between tests to minimize carryover effects.

### Lipid profile and transaminase determination in plasma

Plasma total cholesterol (CHOL HICO GEN.2, #03039773190), triglycerides (TRIGL, #20,767,107,322), alanine transaminase (ALT) (ALTL, #20,764,957,322), and aspartate transaminase (AST) (ASTL, #20,764,949,322) under fasting conditions were measured enzymatically and colorimetrically using commercial kits adapted for the COBAS C501/6000 autoanalyzer (Roche Diagnostics). High-density lipoprotein (HDL) cholesterol levels were determined in plasma obtained after the precipitation of apolipoprotein B (APOB)-containing lipoprotein particles using 0.44 mmol/L phosphotungstic acid (Merck) and 20 mmol/L magnesium chloride (Sigma-Aldrich), followed by enzymatic and colorimetric measurement using the cholesterol reagent adapted for the COBAS C501/6000 autoanalyzer. Very low-density lipoprotein (VLDL) plus low-density lipoprotein (LDL) cholesterol was calculated as the difference between total plasma cholesterol and HDL cholesterol.

### Hematoxylin & eosin and Masson’s Trichrome staining

Both stainings were conducted following standardized protocols. Briefly, the left liver lobe, gonadal white adipose tissue (gWAT), and colon were collected, washed in phosphate-buffer saline, fixed in 4% paraformaldehyde, dehydrated and paraffin embedded. After sectioning at 4 mm, the slides were rehydrated and stained with either hematoxylin and eosin (Agilent/Dako) or Masson’s Trichrome (Agilent/Dako) (only the liver). Samples were then dehydrated, mounted, and observed by light microscopy (Leica DM4 B; MC190 HD, Leica Microsystems). Images were acquired with Leica Application Suite Software.

### Quantification of the percentage of area with steatosis in liver images

Hepatic steatosis in hematoxylin and eosin (H&E) images was quantified using ImageJ. Lipid vacuoles were segmented by applying a fixed threshold (gray intensity values 225–250 on an 8-bit scale), circularity (0.5–1) and restricted area (15–1000 pixel^2^) to avoid artefact counting, including blood vessels, and to include microsteatosis. The percentage of areas with steatosis was then calculated relative to the total tissue area. Three images from three mice from each group were used.

### Adipocyte size and number quantification

Images of gWAT were captured at 20 × magnification and were analyzed using the Adiposoft plug-in for ImageJ-Fiji software to determine adipocyte size. For each mouse, three representative images were evaluated.

### Histiocytes quantification in the liver

Foci of histiocyte quantification in the liver was manually performed by counting the zones with accumulated yellow pigmentation in H&E staining [13] in three photos from three or four mice in each group.

### Colon histological score

Thin coronal sections of colon tissue were examined at 40 × magnification for pathological scoring and assessment of cellular infiltration, following the protocol described by Ahmad et al. [14]. Briefly, sections were graded based on the following criteria: severity of crypt damage, loss of surface epithelium, and spread of inflammation (Supplementary Table 1). In addition, morphometric analysis was performed to measure crypt number, height and width, submucosa thickness, and epithelial weight. Goblet cell abundance within the crypts was also quantified. For each animal, four colon segments (1–2 cm each) were analyzed. Three mice per group were included in the analysis.

### mRNA expression

Tissue samples from liver, adipose tissues (sWAT, gWAT), hypothalamus, and colon (proximal section) were homogenized and lysed with TRIzol® Reagent (Thermo Fisher Scientific). Chloroform (Fisher Scientific) was used for aqueous phase separation, followed by isopropanol (Honeywell) precipitation of RNA, which was washed with 70% ethanol (Sigma-Aldrich) and resuspended in RNase-free water (Fisher Scientific). μDrop Plate and Varioskan Lux Instrument were used to quantify RNA with the Skanit Microplate Reader Software (Thermo Fisher Scientific), and the 260:280 ratio was used to verify RNA purity. RNA was retrotranscribed into cDNA using the High-Capacity cDNA Reverse Transcription Kit (Applied Biosystems). Gene expression was determined by qPCR using the TaqMan Fast Advanced Master Mix and the specific Taqman probes on a Quant Studio 7 Pro Instrument (all from Applied Biosystems). Data were normalized to *B2m* using the comparative Ct method (2^−ΔΔCT^), with relative expression values further normalized to the mean of the CHD male group.

Thermal cycling conditions were as follows: an initial UNG incubation for 2 min at 50ºC, polymerase activation for 1 min at 95ºC, followed by 40 cycles of denaturation at 95ºC for 1 s and annealing/extension at 60ºC for 20 s.

Taqman proves used: *Acaca* (Mm01304257_m1), *Agrp* (Mm00475829_g1) *Iba1* (Mm00479862_g1), *Angptl6* (Mm00513964_m1), *Arg1* (Mm00475988_m1), *Cart* (Mm04210469_m1), *Ccl2* (Mm00441242_31), *Cdh1* (Mm01247357_m1), *Cd36* (Mm004322403_m1), *Cpt1a* (Mm01231183_m1), *Col1a1* (Mm00801666_g1), *Dclk1* (Mm01284845_m1), *Dgat1* (Mm00515643_m1), *Fabp4* (Mm00445878_m1), *Fasn* (Mm00662319_m1), *Fgf21* (Mm07297622_g1), *Fizz1* (Mm00445109_m1), *Gck* (Mm00439129_m1), *Gdf15* (Mm00442228_m1), *Gys2 *(Mm01267381_g1), *Lipe* (Mm00495359_m1), *Igf1* (Mm0439560_m1), *Il1b* (Mm00434228_m1), *Il4ra* (Mm01275139_m1), *Il6* (Mm00446190_m1), *Il10* (Mm01288386_m1), *Il12b* (Mm01288989_m1), *Inhbe* (Mm03023993_m1), *Ldlr* (Mm01177349_m1), *Lep* (Mm00434759_m1), *Mgl1* (Mm00449274_m1), *Mrc1* (Mm01329362_m1), *Muc2* (Mm01276676_m1), *Npy* (Mm01410146_m1), *Nos2* (Mm00440502_m1), *Ocln* (Mm00500910_m1), *Pck1* (Mm01247058_m1), *Pnpla2* (Mm00503040_m1), *Pomc* (Mm00435874_m1) *Pparg* (Mm00440940_m1), *Pygl* (Mm01289790_m1), *Scarb1* (Mm00450234_m1), *Sepp1* (Mm00486048_m1), *Slc2a2* (Mm00446229_m1), *Srebf1* (Mm00550338_m1), *Tgfb1* (Mm01178820_m1), *Tjp1* (Mm01320638_m1), *Tmem119* (Mm00525305_m1), *Tnf *(Mm00443258_m1).

### Western blotting

Liver samples were lysed and homogenized in M-PER buffer (Thermo Scientific) containing Halt Protease and Phosphatase Inhibitor Cocktails (Thermo Scientific). Protein concentration was determined with the BCA Protein Assay Kit (Thermo Scientific). Equal amounts of total protein were separated in SDS–polyacrylamide gel electrophoresis, transferred to Immobilon-P PDVF membranes (Merck Millipore, Burlington, MA) and blocked. Immunoblot analysis was performed with polyclonal antibodies against ACC (3676), HSL (4107), and GS (3893). All antibodies were from Cell Signaling Technology (Danvers, MA) except for a monoclonal antibody against b-ACTIN (Sigma- Aldrich), which was used as loading control. Membranes were incubated with the respective anti-HRP secondary antibodies and visualized with the Immobilon ECL Ultra Western HRP Substrate (Merck Millipore). Band intensity was captured using the iBright CL1000 Imaging System equipped with iBright Analysis Software (Invitrogen, Waltham, MA).

### Statistical analysis

Data were tested for normality using the Shapiro–Wilk test to assess Gaussian distribution. When variables deviated from a normal distribution, appropriate transformations were applied prior to statistical analysis. Outliers were identified using the interquartile range (IQR) method and excluded from analysis when indicated. When data were normally distributed, differences between two groups were assessed using an unpaired two-tailed Student’s *t*-test. For most experiments involving comparisons across sex and diet (CHD vs CDAHFD), two-way ANOVA was performed using sex and diet as independent variables to assess their main effects and interaction, followed by Šídák multiple comparisons post hoc testing. In contrast, for GTT, ITT and PTT tests, two-way ANOVA was conducted using diet and time as independent variables, with Šídák post hoc testing applied to evaluate differences across time points. A *p* < 0.05 was considered statistically significant, while *p values* between 0.05 and 0.07 were considered trends, although not interpreted as statistically significant. In all figures, asterisks (*) denote statistically significant differences between diets, whereas hashtags (#) indicate statistically significant differences between sexes. All symbols reflect the results of post hoc multiple-comparison tests unless otherwise specified. Data are presented as mean ± SEM. GraphPad Prism 10 software for Mac was used to perform all statistical analyses.

## Results

### Female livers display exacerbated early steatosis and distinct transcriptional remodeling under CDAHFD

**Fig. 1:**
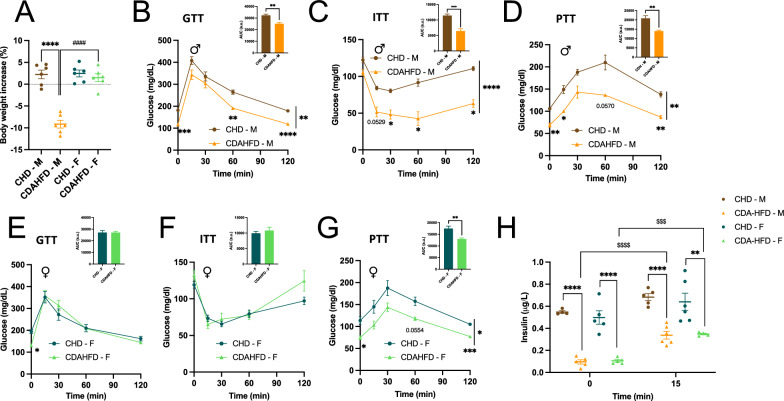
Sex-dependent metabolic adaptations to short-term CDAHFD feeding. After 2 weeks of either chow diet (CHD) or choline-deficient L-amino acid-defined high fat diet (CDAHFD), C57BL/6J male (M) and female (F) mice were assessed for changes in body weight and glucose homeostasis. Body weight increase (**A**). Glucose tolerance test and area under the curve in male mice (**B**). Insulin tolerance test and area under the curve in male mice (**C**). Pyruvate tolerance test and area under the curve in male mice (**D**). Glucose tolerance test and area under the curve in female mice (**E**). Insulin tolerance test and area under the curve in female mice (**F**). Pyruvate tolerance test and area under the curve in female mice (**G**). Circulating insulin levels assessed at baseline and following 15 min of glucose challenge (**H**). Results are presented as mean ± SEM. n = 6 (CHD), n = 5–6 (CDAHFD) per sex. *p < 0.05; **p < 0.01; ***p < 0.001; ****p < 0.0001 (two-way ANOVA (**A**-**H**) and two-tailed unpaired t test (bar graphs)). Differences between sexes are indicated by * and between diets by #

To determine whether sex influences the early stages of MASLD development, we employed a controlled dietary model in which male and female mice were fed either CDAHFD or a control chow diet (CHD) for two weeks, followed by assessment of systemic and hepatic alterations. Notably, male mice on CDAHFD exhibited body weight loss, a phenotype not observed in females (Fig. 1A). Moreover, males displayed improved glucose tolerance (Fig. 1B), insulin sensitivity (Fig. 1C) and reduced gluconeogenic response (Fig. 1D), whereas these metabolic improvements were only partially evident in females, without differences in the GTT and ITT (Fig. 1E, F, G). However, CDAHFD reduced basal insulin levels and improved glucose-stimulated insulin secretion in both sexes (Fig. 1H), indicating that the improved glucose tolerance observed in males is unlikely to be driven by differences in insulin secretion.

**Fig. 2:**
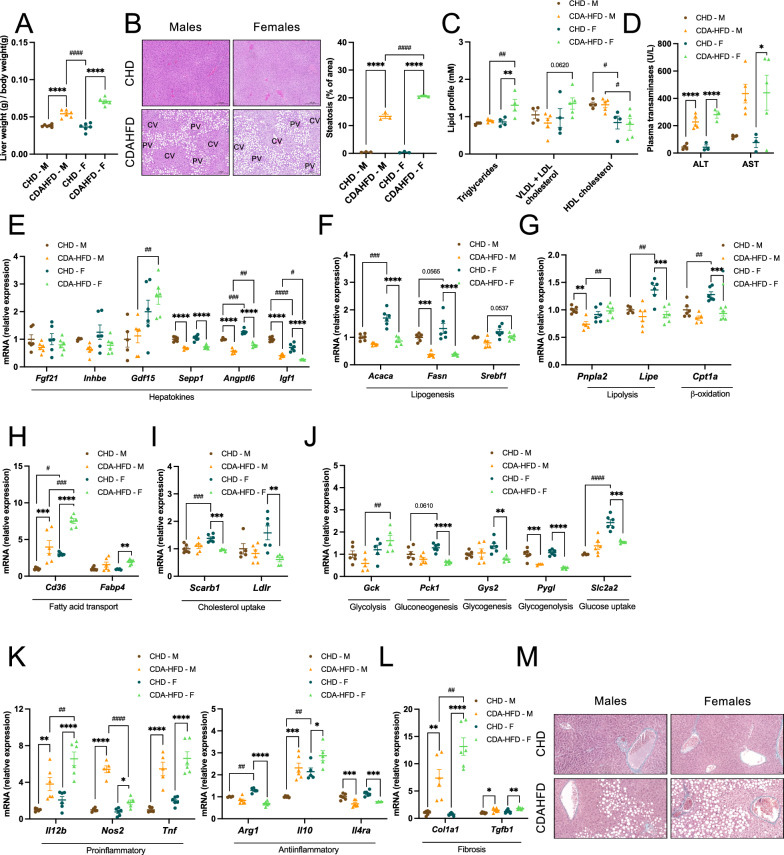
Characterization of sex-specific hepatic alterations after short-term CDAHFD feeding. C57BL/6J male (M) and female (F) mice were subjected to 2 weeks of chow diet (CHD) or choline-deficient L-amino acid-defined high-fat diet (CDAHFD) and the livers and plasma were collected for analysis. Liver weight/body weight ratio (**A**). H&E staining of the liver (scale bars: 200 µm) and % of area with steatosis quantification (**B**). Plasma lipid profile, including triglycerides, very low-density lipoprotein plus low-density lipoprotein (VLDL + LDL) cholesterol and high-density lipoprotein (HDL) cholesterol (**C**). Alanine aminotransferase (ALT) and aspartate aminotransferase (AST) levels in plasma (**D**). RT-qPCR of hepatokines (**E**), lipid metabolism (**F**-**I**), glucose metabolism (**J**), inflammatory (**K**), and fibrosis (**L**) markers in the liver. Hepatic Masson’s trichrome staining (scale bars: 50 µm) (**M**). Numeric results are presented in scatter dot plots as mean ± SEM. n = 4–6 (CHD), n = 5–6 (CDAHFD) per sex. *p < 0.05; **p < 0.01; ***p < 0.001; ****p < 0.0001 (two-way ANOVA (**A**-**L**)). Differences between sexes are indicated by * and between diets by #. CV, central vein; PV, portal vein

Subsequently, we focused our analyses on hepatic outcomes to evaluate sex-dependent susceptibility to develop steatosis and steatohepatitis. Although both males and females showed an increase in liver weight relative to body weight under CDAHFD, the liver/BW ratio was significantly higher in females (Fig. 2A). Accordingly, histological examination (H&E staining) revealed more pronounced steatosis in female livers, characterized by the presence of large periportal macrovesicular lipid droplets together with central microvesicular steatosis, a pattern not observed in males (Fig. 2B).

Plasma lipid profiling further supported the exacerbated phenotype in females. CDAHFD significantly increased plasma triglyceride levels in females, which were also higher than those observed in males under the same dietary condition (Fig. 2C). In addition, a trend toward higher VLDL/LDL cholesterol levels was observed in females compared with males under CDAHFD (p = 0.0620; Fig. 2C). In contrast, HDL cholesterol levels were not affected by the diet; however, females consistently displayed lower HDL levels than males, regardless of dietary condition (Fig. 2C). Plasma ALT levels increased in response to CDAHFD in both sexes, whereas AST levels reached statistical significance only in females, despite a similar upward trend in males (Fig. 2D).

At the transcriptional level, two weeks of CDAHFD feeding induced a coordinated regulation of hepatokines that was largely independent of sex in terms of dietary response. In both males and females, CDAHFD provoked a marked downregulation of *Sepp1*, *Angptl6*, and *Igf1* (Fig. 2E)*,* indicating activation of endocrine and metabolic stress pathways in response to the nutritional challenge. Although the magnitude of diet-induced downregulation was comparable between sexes, *Angptl6* and *Igf1* displayed sex-specific differences in basal expression levels under chow-fed conditions. Remarkably, hepatic *Gdf15* mRNA expression, an hepatokine linked to MASLD [15], was significantly elevated in CDAHFD-fed females compared with diet-matched males (Fig. 2E), suggesting it may contribute to their exacerbated early hepatic phenotype.

Sex-dependent divergence was also evident in the regulation of hepatic metabolic pathways after two weeks of CDAHFD, with females and males engaging in distinct programs of lipid and glucose handling (Fig. 2F-J). Under chow-fed conditions, females displayed higher hepatic expression of *Acaca* compared with males, which was markedly reduced following CDAHFD feeding, indicating a sex-specific regulation of this lipogenic enzyme (Fig. 2F). In contrast, *Fasn* expression was similarly downregulated by CDAHFD in both sexes, suggesting a shared dietary response, while *Srebf1* exhibited only modest changes without clear sex-dependent differences (Fig. 2F). In males, CDAHFD selectively reduced hepatic *Pnpla2* expression, consistent with reduced lipolysis in response to the diet (Fig. 2G). Conversely, females exhibited a coordinated downregulation of *Lipe* and *Cpt1a* upon CDAHFD feeding while showing higher basal expression levels (Figs. 2G), consistent with attenuated lipid mobilization and fatty acid catabolism in this sex. Regarding lipid uptake, *Cd36* expression increased in response to CDAHFD in both sexes, but reached significantly higher levels in females, whereas *Fabp4* was selectively induced in females in response to CDAHFD (Fig. 2H). In contrast, markers of hepatic cholesterol uptake (*Scarb1* and *Ldlr*), which were elevated in chow-fed females, were reduced following CDAHFD in this sex (Fig. 2I), consistent with impaired hepatic cholesterol handling.

This remodeling of lipid metabolism was paralleled by changes in glucose-related genes. *Pck1* and *Slc2a2*, which were more highly expressed in chow-fed females, were downregulated by CDAHFD, suggesting reduced gluconeogenesis and glucose uptake, respectively (Fig. 2J). *Gys2* was also selectively downregulated in females, suggesting decreased glycogenesis, while *Pygl* was similarly modulated in both sexes. Although *Gck* was not significantly regulated by diet in either sex, its expression was higher in CDAHFD-fed females compared with diet-matched males, consistent with enhanced glycolytic capacity. Several of these transcriptional changes were reflected at the protein level. Consistent with mRNA data, hepatic HSL protein (encoded by *Lipe*) levels were significantly reduced in CDAHFD-fed females (Supplementary Fig. 1), supporting a suppression of lipolytic pathways in this sex. In contrast, ACC (encoded by *Acaca*) and GS (encoded by *Gys2*) protein levels were reduced in both sexes (Supplementary Fig. 1), indicating that some diet-induced effects on lipid and glycogen synthesis extend beyond transcriptional regulation and are not strictly sex-specific.

Pro- and anti-inflammatory markers exhibited broadly similar diet-induced trends in both sexes, characterized by an upregulation of pro-inflammatory genes and a general downregulation of anti-inflammatory ones, with *Il10* representing a notable exception (Fig. 2K). Notably, *Arg1* expression was selectively downregulated in females following CDAHFD, likely reflecting sex-specific regulation influenced by their higher basal expression levels. Fibrotic markers, including *Col1a1* and *Tgfb1*, were induced in response to CDAHFD in both sexes; however, CDAHFD-fed females reached significantly higher *Col1a1* expression levels compared with diet-matched males (Fig. 2L). Despite these transcriptional changes, Masson’s trichrome staining did not reveal evident collagen deposition beyond the perivenular and periportal regions in either group (Fig. 2M), indicating that two weeks of CDAHFD feeding does not result in histologically detectable fibrosis.

Although females are considered relatively protected from MASLD progression, the temporal dynamics of this protection remain unclear. Analysis of systemic and hepatic responses after prolonged CDAHFD exposure (14 weeks) revealed a preserved hepatic phenotype in female mice, consistent with sex-dependent disease susceptibility [4, 5]. CDAHFD-fed females did not exhibit body weight loss, although their weight gain relative to chow-fed controls was minimal, whereas male mice under CDAHFD displayed weight loss, with approximately 30% lower weight gain compared with sex-matched controls (Supplementary Fig. 2A). Hepatic transcriptomic analysis revealed sex-specific alterations in the expression of genes involved in lipid and glucose metabolism (Supplementary Fig. 2B-F), showing both similarities and differences compared with short-term CDAHFD exposure.

Most proinflammatory markers were induced in response to the diet in both sexes. However, significantly higher expression levels of *Il12b* and *Tnf* were observed in males*,* while *Nos2* was selectively modulated in this sex (Supplementary Fig. 2G). Anti-inflammatory markers were largely unchanged, except for *Arg1*, which was downregulated exclusively in females (Supplementary Fig. 2H). Consistent with these findings, fibrotic markers were more strongly elevated in males, and *Tgfb1* showed significant upregulation only in this sex (Supplementary Fig. 2I). Accordingly, histological analyses further revealed reduced histiocyte accumulation and qualitatively lower collagen deposition in female livers, despite comparable levels of hepatic steatosis (Supplementary Fig. 2 J-K).

In line with these observations, male mice fed a CDAHFD showed a blunted pyruvate-induced glucose production (Supplementary Fig. 2L), whereas females exhibited a preserved gluconeogenic response compared to their sex-matched controls (Supplementary Fig. 2M). Taken together, these findings indicate that prolonged CDAHFD exposure induces a worsened phenotype in males, whereas short-term feeding elicits distinct sex-dependent hepatic adaptations, with females exhibiting an exacerbated steatotic and metabolic response.

### Early-phase hypothalamic adaptations to CDAHFD exposure differ by sex

**Fig. 3:**
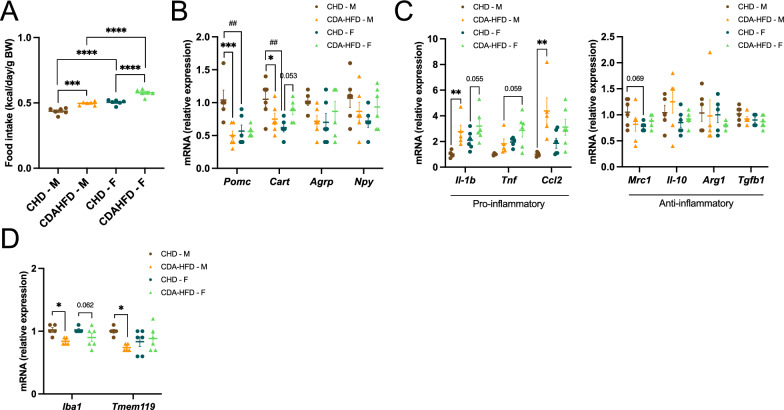
Early sex-dependent hypothalamic responses to short-term CDAHFD. Following 2 weeks of chow diet (CHD) or choline-deficient L-amino acid-defined high-fat diet (CDAHFD) feeding, hypothalamic samples were collected from male (M) and female (F) mice for subsequent analyses. Average food intake normalized by body weight (**A**). RT-qPCR analysis of hypothalamic appetite-regulating neuropeptides (**B**), markers of inflammation (**C**) and microglial homeostasis (**D**). Results are presented as mean ± SEM. n = 4–6 (CHD), n = 4–6 (CDAHFD) per sex. *p < 0.05; **p < 0.01; ***p < 0.001; ****p < 0.0001 (two-way ANOVA for all graphs). Differences between sexes are indicated by * and between diets by #

Given the emerging role of the brain-liver axis in metabolic disease, we next examined whether early adaptive phase hypothalamic responses to CDAHFD might contribute to the observed sexual dimorphism in MASLD progression. CDAHFD increased food intake when normalized to body weight in both sexes, with females showing the highest relative intake overall (Fig. 3A); however, in males this increase in relative intake coincided with a reduction in body weight (Fig. 1A), rather than reflecting an absolute increase of food consumption, as total caloric intake was unchanged between diets (data not shown).

Under CHD conditions, males exhibited higher *Pomc* and *Cart* mRNA levels than females, indicating basal sexual dimorphism in anorexigenic hypothalamic signaling (Fig. 3B). CDAHFD feeding resulted in a marked reduction of *Pomc* expression in males, with a similar diet-induced decrease observed for *Cart*, whereas expression levels in females were largely preserved. In contrast, the orexigenic transcripts *Agrp* and *Npy* did not show significant differences across diets or sexes (Fig. 3B). Together, these data indicate that short-term CDAHFD selectively suppresses anorexigenic hypothalamic signaling in males under fasting conditions, while females maintain a relatively stable anorexigenic profile.

We next assessed the hypothalamic inflammatory tone. Classical pro-inflammatory cytokines genes showed increased levels in response to CDAHFD, with males generally exhibiting higher response than females (Fig. 3C). In contrast, anti-inflammatory markers were not significantly altered by diet or sex. In parallel, expression of microglial homeostatic markers revealed clear sex-dependent alterations, with both *Iba1* and *Tmem119* reduced in CDAHFD-fed males compared with CHD controls (Fig. 3D). Together, these data indicate that short-term CDAHFD promotes a sex-dependent shift towards a pro-inflammatory hypothalamic transcriptional profile, particularly in males, characterized by reduced expression of microglial homeostatic markers and occurring in the absence of a compensatory anti-inflammatory response, which is consistent with early microglial activation that is not observed in females.

After 14 weeks of dietary intervention, hypothalamic alterations preserved the sex-dependent pattern observed during the short-term period, although specific transcriptional signatures differed over time. Differences in food intake persisted at 14 weeks when normalized to body weight, with CDAHFD-fed mice, especially females, displaying higher relative intake than CHD counterparts (Supplementary Fig. 3A); however, this pattern reflected sustained differences in body weight rather than an increase in absolute food consumption. Hypothalamic neuropeptide expression exhibited substantial inter-individual variability, with no consistent diet-dependent changes across *Pomc*, *Cart*, *Agrp* or *Npy*, indicating the absence of a uniform long-term transcriptional shift in core appetite-regulating pathways (Supplementary Fig. 3B). In contrast, hypothalamic inflammatory markers revealed clearer sex-dependent effect at this later stage. CDAHFD-fed male mice showed elevated expression of pro-inflammatory genes, most notably *Il-1b* and *Tnf*, compared to CHD-fed male controls, with no corresponding induction observed in females (Supplementary Fig. 3C). Expression of anti-inflammatory markers remained largely unchanged across groups, consistent with a modest pro-inflammatory bias rather than a broad activation of alternative inflammatory programs (Supplementary Fig. 3C). Consistently, homeostatic microglial markers were downregulated in CDAHFD-fed males, aligning with the observed pro-inflammatory shift (Supplementary Fig. 3D). Therefore, long-term CDAHFD feeding elicits similar sex-dependent effects in pro-inflammatory and microglial markers to the ones observed in short-term feeding, whereas neuropeptide expression becomes unmodulated. 

### Adipose tissue shows limited sex-specific responses to CDAHFD

**Fig. 4:**
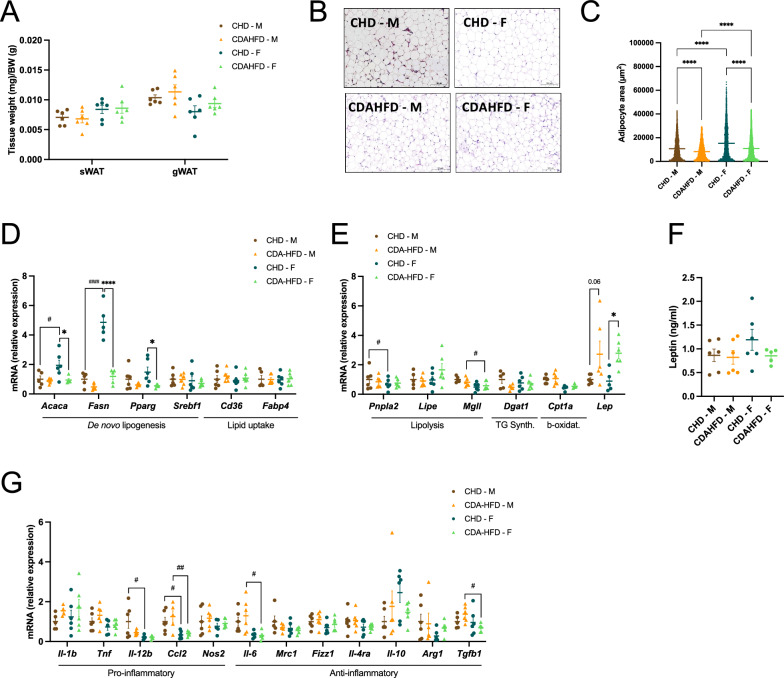
Adipose tissue adaptations to short-term CDAHFD feeding in male and female mice. Male (M) and female (F) mice were exposed to chow diet (CHD) or choline-deficient L-amino acid-defined high-fat diet (CDAHFD) for 2 weeks prior tissue evaluation. Subcutaneous (sWAT) and gonadal (gWAT) white adipose tissue weights in males and females (**A**). H&E staining of the gonadal white adipose tissue (scale bars: 100 µm) (**B**). Quantification of adipocyte area (**C**). RT-qPCR analysis of lipid metabolism (**D**, **E**) in gonadal white adipose tissue. Fasting circulating leptin levels (**F**). RT-qPCR analysis of inflammatory markers (**G**) in gonadal white adipose tissue. Numeric results are presented as mean ±  SEM. n = 4–6 (CHD), n = 4–6 (CDAHFD) per sex. *p < 0.05; **p < 0.01; ***p < 0.001; ****p < 0.0001 (two-way ANOVA for all graphs). Differences between sexes are indicated by * and between diets by #

To evaluate peripheral metabolic adaptations to short-term CDAHFD feeding, we performed a comprehensive analysis of white adipose tissue (WAT) mass, morphology, and transcriptional profile. Neither subcutaneous (sWAT) nor gWAT depot weights differed significantly between dietary groups in either sex (Fig. 4A). In contrast, histological assessment revealed significant alterations in adipocyte morphology, with CDAHFD feeding inducing a reduction in adipocyte area in both males and females, indicating diet-induced adipocyte remodeling at this early stage (Fig. 4B-C).

At the transcriptional level, CDAHFD feeding induced sex-dependent changes in adipose lipid metabolic pathways. Genes involved in *de novo* lipogenesis, including *Acaca*, *Fasn*, and *Pparg*, were significantly downregulated in females (Fig. 4D), consistent with their higher expression levels under chow-fed conditions, whereas expression of lipid uptake-related genes (*Cd36*, *Fabp4*) remained largely unchanged. Analysis of lipid turnover pathways further revealed sex-dependent differences in the expression of select lipolytic genes (Fig. 4E). Under CHD conditions, females exhibited lower *Pnpla2* expression compared with males, while under CDAHFD feeding, *Mgll* expression was significantly reduced in females relative to male counterparts. In contrast, genes involved in triglyceride synthesis (*Dgat1*) and b-oxidation (*Cpt1a*) were not significantly altered across diets or sexes (Fig. 4E).

Adipose-derived leptin expression showed diet-dependent regulation at the transcriptional level. *Lep* mRNA expression was increased in CDAHFD-fed mice compared with chow-fed controls, reaching statistical significance in females (Fig. 4E). However, circulating plasma leptin concentrations did not differ among experimental groups (Fig. 4F).

Inflammatory response revealed selective sex-dependent differences across dietary conditions (Fig. 4G). Under CHD feeding, females exhibited significant lower expression of pro-inflammatory marker *Il12b* compared with males. Under CDAHFD feeding, females displayed significantly reduced expression of *Ccl2* and *Il-6* relative to CDAHFD-fed males. Anti-inflammatory markers were largely unchanged across diets and sexes, although *Tgfb1* expression was significantly reduced in females compared to males under CDAHFD feeding (Fig. 4G).

Together, these data indicate that short-term CDAHFD feeding has minimal impact on adipose tissue mass, with most observed changes reflecting pre-existing baseline sex differences. The diet induces modest adipocyte remodeling and drives sex-dependent transcriptional adaptations in lipid metabolism and inflammatory signaling, with females showing somewhat stronger suppression of inflammatory gene expression and greater modulation of lipid metabolic pathways.

### Females exhibit severe colon tissue remodeling in response to CDAHFD

**Fig. 5:**
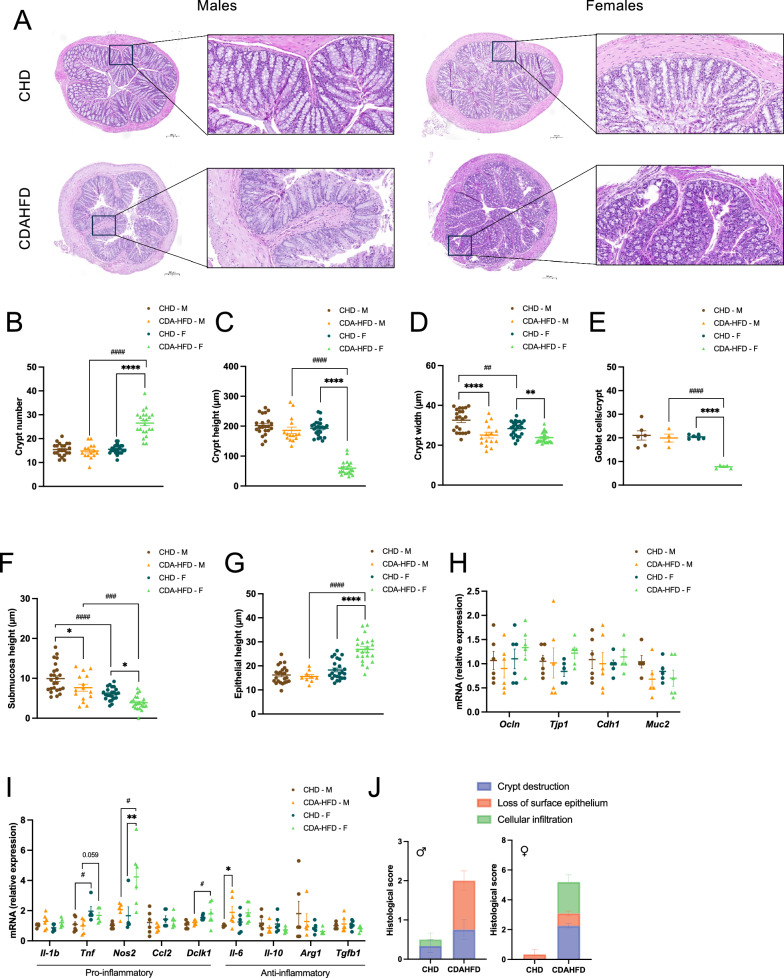
Histological evaluation of colon morphology in control and CDAHFD-fed mice. Following 2 weeks of chow diet (CHD) or choline-deficient L-amino acid-defined high-fat diet (CDAHFD) feeding, colon samples from male (M) and female (F) mice were obtained. H&E-stained colon sections (scale bars: 200 µm for overview; 50 µm for magnified images) (**A**). Quantification of crypt number (**B**). Assessment of crypt height (**C**) and width (**D**). Number of goblet cells per crypt (**E**). Quantification of submucosa (**F**) and epithelial (**G**) height. RT-qPCR analysis of intestinal barrier-related (**H**) and inflammatory markers expression (**I**). Histological score of colonic sections (**J**). Numeric results are presented as mean ±  SEM. n = 4–6 (CHD), n = 4–6 (CDAHFD) per sex. *p < 0.05; **p < 0.01; ***p < 0.001; ****p < 0.0001 (two-way ANOVA for all graphs). Differences between sexes are indicated by * and between diets by #

Building on the critical influence of the gut-liver axis in MASLD progression [16], we finally evaluated colon morphology to explore potential sex-specific intestinal alterations. Histological H&E staining revealed distinct morphological differences between CDAHFD-fed mice and control, with alterations markedly more pronounced in females, which clearly showed atrophied crypts (Fig. 5A). Indeed, female mice fed a CDAHFD exhibited an increased number of crypts (Fig. 5B), characterized by reduced height (Fig. 5C) and width (Fig. 5D), alongside fewer goblet cells per crypt (Fig. 5E). These changes were further associated with reduced submucosa height (Fig. 5F) and increased epithelial height (Fig. 5G). Correspondingly, female CDAHFD mice displayed trends towards elevated expression of gut barrier-related genes (Fig. 5H) and statistically significant heightened inflammatory markers, compared to both CHD controls and male mice (Fig. 5I). In contrast, CDAHFD-fed male mice displayed milder alterations, primarily limited to reduced crypt width (Fig. 5D), decreased submucosa height (Fig. 5F) and attenuated inflammatory response (Fig. 5I). CDAHFD induced elevated levels of colonic structural and functional alterations in both sexes, with female mice exhibiting the most pronounced changes, reflected in the highest overall histological score (Fig. 5J). Conversely, upon prolonged CDAHFD exposure (14 weeks), females displayed a reversion of the earlier exacerbated intestinal alterations, resulting in comparable intestinal barrier integrity (Supplementary Fig. 4A) and inflammatory profiles (Supplementary Fig. 4B) between sexes.

Overall, these findings demonstrate a marked sex-dependent intestinal response to CDAHFD. Females exhibit acute and pronounced colon remodeling, while males show only mild structural alterations and a blunted inflammatory response, indicating relative resistance of the colon to early diet-induced stress. Importantly, these differences diminish with prolonged CDAHFD exposure, suggesting that intestinal dysfunction is an early and transient feature predominantly affecting females. These observations reveal the gut as a key site of early sexual dimorphism in MASLD pathogenesis.

## Discussion

MASLD exhibits a well-documented sexual dimorphism in both humans and preclinical models, traditionally characterized by a delayed progression in females until menopause [4, 5, 7, 8, 17, 18]. However, the early sex-dependent mechanisms that precede advanced liver pathology remain insufficiently defined due to the predominant use of mid- to long-term dietary interventions [17]. By examining an early time point in the CDAHFD model, before overt fibrosis or systemic deterioration, we uncovered unanticipated temporal sexual dimorphism, revealing that disease susceptibility is initially greater in females despite their classical long-term protection. These findings underscore that sex differences in MASLD are dynamic rather than static, and highly dependent on disease stage and experimental context.

Our results demonstrate a clear sex-specific response to CDAHFD. Only male mice exhibited body weight loss and improved glucose tolerance, whereas both basal and glucose-stimulated insulin secretion were improved in both sexes, suggesting that insulin-dependent mechanisms are unlikely to account for the observed dimorphism. Male-biased body weight loss has been reported in similar dietary interventions [10, 12, 19, 20], but is generally interpreted as a consequence of systemic metabolic stress rather than a beneficial metabolic adaptation. Mechanistically, choline deficiency limits hepatic VLDL synthesis and secretion [21], while methionine restriction further impairs VLDL production while modulating oxidative stress [22]. In addition, methionine restriction has been shown to enhance energy expenditure and limit fat deposition, contributing to weight loss, as reported for MCD and methionine-restricted diets [23–27] In contrast, female mice preserved body weight under CDAHFD exposure, but this was dissociated from improvements in glucose homeostasis and coincided with impaired hepatic lipid handling, suggesting divergent adaptive versus maladaptive metabolic trajectories between sexes or diets. Adipose tissue depots and skeletal muscle mass (data not shown), did not differ between sexes or diets, suggesting that these tissues alone are unlikely to account for the observed sex differences in body weight. A limitation of the present study is the absence of comprehensive body composition analyses; precluding definitive conclusions regarding the relative contribution of fat mass, lean mass or water content to the sex-specific changes in body weight. Although greater fat mass loss has been reported in male mice subjected to methionine-restricted diets [27], it remains unclear whether similar mechanisms apply to our CDAHFD model, which combines choline deficiency and methionine restriction and may affect energy balance and tissue composition differently.

In the liver, female mice developed an exacerbated phenotype after only two weeks of CDAHFD, consistent with greater hepatic lipid accumulation and increased liver-to-body weight ratio. Sex-dependent differences in short MASLD-inducing dietary interventions have been scarcely explored and show discrepant results compared with our findings [28, 29], supporting the notion that early sexual dimorphism in MASLD is highly dependent on dietary context, specifically the degree of methionine restriction. Metabolic variability across C57BL/6 substrains may further contribute to these differences [28], highlighting an inherent limitation of comparing artificial MASLD-inducing diets and using mice with different genetic backgrounds, which can differentially engage pathogenic pathways and yield divergent sex-dependent outcomes. Mechanistically, early hepatic stress in females was associated with higher expression of *Gdf15,* an hepatokine elevated in MASLD patients compared with healthy controls^15^. Although females suppressed transcriptional programs of lipogenesis (*Acaca*, *Fasn*) and cholesterol uptake (*Scarb1*, *Ldlr*), the concomitant reduction in lipolysis (*Lipe*) and β-oxidation (*Cpt1a*), together with increased fatty acid uptake (*Cd36*, *Fabp4*) suggests that inhibition of lipogenesis alone is insufficient to compensate for lipid overload. In contrast, male livers displayed a more limited transcriptional response, consistent with greater early metabolic flexibility in males, enabling transient protection against hepatic lipid accumulation.

After prolonged CDAHFD exposure, the pattern of sex differences shifted, with male mice developing greater hepatic inflammation and fibrosis, consistent with previous findings [17, 18]. The progressive deterioration in males aligns with clinical MASLD progression, where men exhibit earlier disease onset and faster progression to fibrosis [4, 5]. Several mechanisms may contribute to relative long-term female protection, including estrogen modulation of choline metabolism, via induction of phosphatidylethanolamine N-methyltransferase (PEMT), which enhances endogenous choline synthesis [30]. This may partially compensate for choline deficiency and reduced VLDL export associated to CDAHFD [21] and could explain the divergent outcomes observed in other diet-induced MASH models [31]. Additionally, estrogens suppress NF-κB-driven inflammation [32, 33], consistent with the lower hepatic expression of *Il12b* and *Tnf* detected in females. Nevertheless, as circulating estrogen levels were not measured and ovariectomy-based interventions were not performed, the contribution of estrogens to these sex-dependent effects cannot be directly established in this study.

To assess whether central mechanisms contribute to the early sex-dependent metabolic trajectories, we analyzed hypothalamic regulatory pathways. Males exhibited a selective suppression of anorexigenic neuropeptides, including *Pomc* and *Cart*, whereas females largely preserved anorexigenic signaling, despite more pronounced peripheral remodeling. These central alterations occurred despite increased food intake normalized to body weight in both sexes, while only males exhibited lower body weight, indicating an uncoupling between energy intake and body mass regulation. This pattern indicates that, during early CDAHFD exposure, males may experience a transient negative energy balance or reduced energetic efficiency, consistent with their improved glucose tolerance. Rather than reflecting a metabolically advantageous state, this pattern likely represents an early adaptive or catabolic response to diet-induced hepatic stress. However, as energy expenditure was not directly measured, altered energy dissipation cannot be excluded and represents a limitation of the study.

Leptin and insulin are key regulators of hypothalamic energy sensing and inflammatory signaling, with documented sex-dependent differences in their central actions [34, 35]. However, circulating leptin and insulin levels did not differ markedly between sexes in our model, suggesting that the divergent hypothalamic are more likely driven by differences in central sensitivity or downstream signaling rather than systemic hormone availability.

Early CDAHFD exposure also elicited sex-divergent hypothalamic inflammatory responses. Males showed reduced expression of microglial-associated markers, including *Iba1* and *Tmem119*, whereas females showed a comparatively stable transcriptional profile with attenuated induction of pro-inflammatory genes, suggesting differential neuroimmune adaptation between sexes. Hypothalamic inflammatory and stress-related pathways have been shown to modulate systemic glucose and lipid metabolism in response to nutritional challenges [36–39], particularly under conditions of energy imbalance. In this context, the reduction of microglial-associated transcripts in males may reflect an early central adjustment to metabolic stress preceding overt neuroinflammatory activation. However, in the absence of direct functional or compositional analyses of the hypothalamus, causal links between these transcriptional changes and peripheral metabolic outcomes remain speculative.

Adipose tissue also displayed sex-dependent adaptations to short-term CDAHFD exposure, although these changes were less pronounced than those observed in the liver, suggesting that white adipose tissue is not the primary early driver of sex divergence in MASLD but may contribute to its progression. Adipose depot mass was preserved in both sexes, while both males and females showed a reduction in adipocyte size, consistent with enhanced lipid mobilization, as previously reported under methionine restriction and MCD feeding [25–27]. Similar reductions in adipocyte size have been described in response to short-term dietary stress and are thought to precede overt changes in adipose tissue mass or inflammation [40]. At the transcriptional level, CDAHFD induced sex-dependent changes in adipose lipid metabolism that largely reflected baseline differences between males and females, rather than coordinated diet-driven reprogramming. In females, downregulation of lipogenic genes occurred in the context of higher basal expression under chow-fed conditions, suggesting convergence towards a shared metabolic state. Endocrine and inflammatory outputs from adipose tissue were only modestly affected, and despite increased leptin mRNA expression, circulating leptin levels remained unchanged. Consistent with reports that adipose inflammatory remodeling is a later event in diet-induced metabolic disease [40, 41], inflammatory gene expression showed limited sex-dependent differences. Together, these findings suggest that adipose tissue is unlikely to drive early systemic or central phenotypes under CDAHFD and instead provides a permissive metabolic context during the initial dietary challenge.

A major novel contribution of this study is the demonstration that tissue responses to metabolic challenge are temporally and spatially dissociated rather than synchronous. We identified clear organ-specific sensitivity to CDAHFD, with the colon emerging as one of the earliest and most diet-sensitive tissues. Two weeks of CDAHFD were sufficient to induce pronounced crypt hyperplasia and remodeling, loss of goblet cells, epithelial hypertrophy, and reduced submucosal thickness in females, consistent with an epithelial repair-biased response associated with compromised mucosal protection. Similar sex-dependent disturbances in intestinal morphology have been described under high-fat diets [42] and in intestine-specific *Fmo5* knockout mice [43], supporting the notion that intestinal susceptibility to metabolic stress is sexually dimorphic. Combined with increased intestinal inflammatory gene expression, these findings suggest early compromise of intestinal barrier integrity in females. Such alterations have been mechanistically linked to hepatic lipid deposition and inflammation via gut-derived endotoxemia and increased portal exposure to microbial products [16]. In line with this interpretation, previous studies have documented sex differences in intestinal microbiota composition and metabolite profiles in rodents with MASLD [44–46], suggesting a sex-dependent contribution of the gut–liver axis to early disease pathogenesis.

Although intestinal responses at later stages were assessed only at the transcriptional level, sex differences in barrier- and inflammation-related gene expression were no longer evident, consistent with attenuation of the early molecular alterations observed at short-term CDAHFD exposure. Nevertheless, the absence of histological analysis at this stage precludes conclusions regarding structural recovery of the colon.

Collectively, our data indicate that sex-related differences in MASLD emerge early and evolve dynamically across disease stages. Females exhibit a pronounced early response to metabolic and intestinal stress, whereas males engage adaptive mechanisms that transiently sustain metabolic balance, accompanied by sex-dependent hypothalamic adaptations consistent with altered central energy and inflammatory signaling. At later stages, this pattern reverses, with males developing a more pronounced inflammatory and fibrotic phenotype in the liver. These findings highlight the dynamic and system-wide nature of sexual dimorphism in MASLD. From a translational perspective, while these sex-specific trajectories may be relevant to human disease, their extrapolation warrants caution given the contribution of dietary context and inter-individual genetic variability to clinical MASLD.

### Perspectives and significance

This study reveals that sexual dimorphism in MASLD is dynamic, with females exhibiting early vulnerability before transitioning to a classically protective phenotype. These finding highlight the importance of temporal resolution when studying sex differences in metabolic liver disease and suggest that early-phase mechanisms, rather than late-stage pathology, may set the trajectory for divergent disease outcomes. The sex-specific neuroimmune and gut-liver adaptations identified here raise the possibility that early communication between the intestine, liver and brain contributes to later resilience or susceptibility. Understanding how these circuits evolve across time and hormonal status may unlock precision-tailored therapeutic windows in which early interventions could reverse or reprogram disease trajectories in a sex-dependent manner. Future work integrating hormonal modulation, intestinal microbiota profiling, and neuroendocrine regulation will be essential to fully elucidate the inter-organ mechanisms governing sex-dependent MASLD progression.

## Supplementary Information

Additional file 1.

## Data Availability

All data generated or analyzed during this study are included within the manuscript and supporting documents.
