# Supplementary material for: Sex-specific dynamics of MASLD reveal early hepatic and extrahepatic metabolic deterioration in females despite long-term protection
Source: Biol Sex Differ. 2026 Mar 22;17:92. doi: 10.1186/s13293-026-00876-y (PMC13130735; doi:10.1186/s13293-026-00876-y)
Supplement: Supplementary file 1 — Additional file 1. [file 13293_2026_876_MOESM1_ESM.pdf]

Supplementary material

Sex-specific dynamics of MASLD reveal early hepatic and extrahepatic metabolic deterioration in females despite long-term protection

Repollés-de-Dalmau M[1, 2, 3]*; Marsal-Beltran A[1, 2, 3]*; Núñez-Roa C[1, 2]; Vendrell J[1, 2, 3]; Ceperuelo-Mallafré V[1, 2, 3]; Fernández-Veledo S[1, 2, 3]

[1] DIAMET Research Group, Institut de Recerca Biomèdica Catalunya Sud (formerly Institut d'Investigació Sanitària Pere Virgili); Hospital Universitari Joan XXIII, Tarragona 43005, Spain.

[2] CIBER de Diabetes y Enfermedades Metabólicas Asociadas (CIBERDEM), Instituto de Salud Carlos III, Madrid 28029, Spain

[3] Rovira i Virgili University, Reus 43201, Spain

*These authors contributed equally.

Correspondence

Address correspondence to: Sonia Fernández Veledo, PhD. Institut de Recerca Biomèdica Catalunya Sud, C/ Dr. Mallafré Guasch 4, Tarragona 43005 (Spain). sonia.fernandez@irbcatsud.cat; tel: +34 977 295 800 ext. 3401.

Supplementary Table 1. Colon histomorphology and neutrophil infiltration scoring criteria

Category	Histological criterion	Score Value
Crypts	Intact crypts	0
	Disoriented crypts	1
	Variable crypts diameter	2
	Atrophied crypts	3
	Mucosa devoid of crypts	4
Loss of surface epithelium	Intact surface epithelium	0
	Sloughing off epithelial surface	1
	Patchy loss of surface epithelium	2
	Moderate loss of surface epithelium	3
	Severe loss/erosion of surface epithelium	4
Cellular infiltration	Clear mucosa/submucosa	0
	Mucosal/lamina propria infiltration	1
	Mucosal and submucosal infiltration	2
	Moderate cryptitis/infiltration to crypt epithelial cells	3
	Severe cryptitis	4

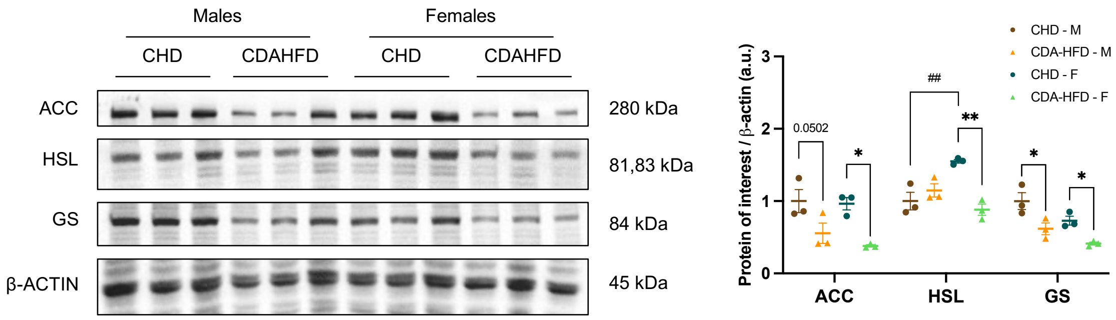

Supplementary Figure 1. Hepatic protein expression analysis following short-term CDAHFD feeding. Following 2 weeks of chow diet (CHD) or choline-deficient L-amino acid-defined high-fat diet (CDAHFD) feeding, liver samples were collected from male (M) and female (F) mice for subsequent analyses. Representative Western blot images showing hepatic protein levels (left) and densitometric quantification of protein abundance normalized to b-actin (right). Data are presented as mean ± SEM (n= 3/group). *$p<0.05$; **$p<0.01$ (two-way ANOVA). Differences between sexes are indicated by * and between diets by #.

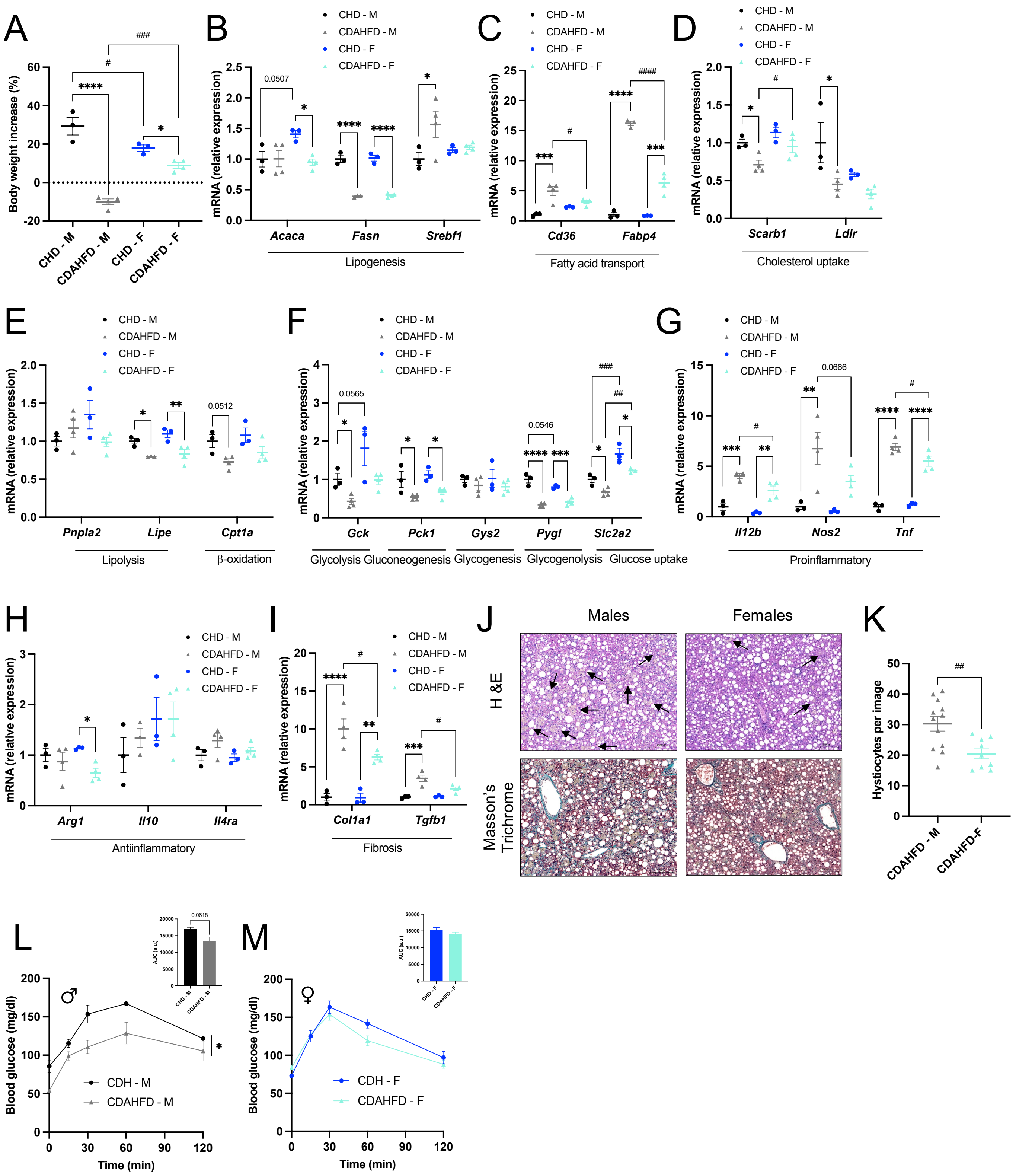

A
Body weight increase (%)
CHD - M
CDAHFD - M
CHD - F
CDAHFD - F
****
*
#
###
B
CHD - M
CDAHFD - M
CHD - F
CDAHFD - F
mRNA (relative expression)
0.0507
Acaca
Fasn
Srebf1
Lipogenesis
C
Cd36
Fabp4
Fatty acid transport
####
D
Scarb1
Ldlr
Cholesterol uptake
E
Pnpla2
Lipe
Cpt1a
0.0512
Lipolysis
β-oxidation
F
0.0565
0.0546
Gck
Pck1
Gys2
Pygl
Slc2a2
Glycolysis
Gluconeogenesis
Glycogenesis
Glycogenolysis
Glucose uptake
##
G
0.0666
Il12b
Nos2
Tnf
Proinflammatory
H
Arg1
Il10
Il4ra
Antiinflammatory
I
Col1a1
Tgfb1
Fibrosis
J
Males
Females
H &E
Masson's Trichrome
K
Hystiocytes per image
CDAHFD - M
CDAHFD-F
L
Blood glucose (mg/dl)
Time (min)
CDH - M
CDAHFD - M
AUC (a.u.)
0.0618
♂
M
CDH - F
CDAHFD - F
♀

Supplementary Figure 2. Sex-dependent liver alterations after 14-week CDAHFD exposure. Male (M) and female (F) mice were subjected to 14 weeks of chow diet (CHD) or choline-deficient L-amino acid-defined high-fat diet (CDAHFD) feeding, and the livers were harvested for further analysis. Body weight increase percentage (**A**). Gene expression analysis of lipid (**B-E**) and glucose metabolism (**F**) markers in the liver. mRNA expression of hepatic inflammatory (**G**-**H**) and fibrotic markers (**I**). H&E (top panels) and Masson's trichrome (bottom panels) staining in the livers of CDAHFD-fed animals (**J**). Histiocyte quantification in H&E images of the livers in CDAHFD-fed animals (**K**). Pyruvate tolerance test at week 13 of male (**L**) and female (**M**) mice. Numeric results are presented as mean ± SEM (n= 3 CHD/sex; 3-4 CDAHFD/sex). *p<0.05; **p<0.01; ***p<0.001; ****p<0.0001 (two-way ANOVA (**A-I; L-M**), two-tailed unpaired t-test or Mann-Whitney test (**K** and bar graphs). Differences between sexes are indicated by * and between diets by #.

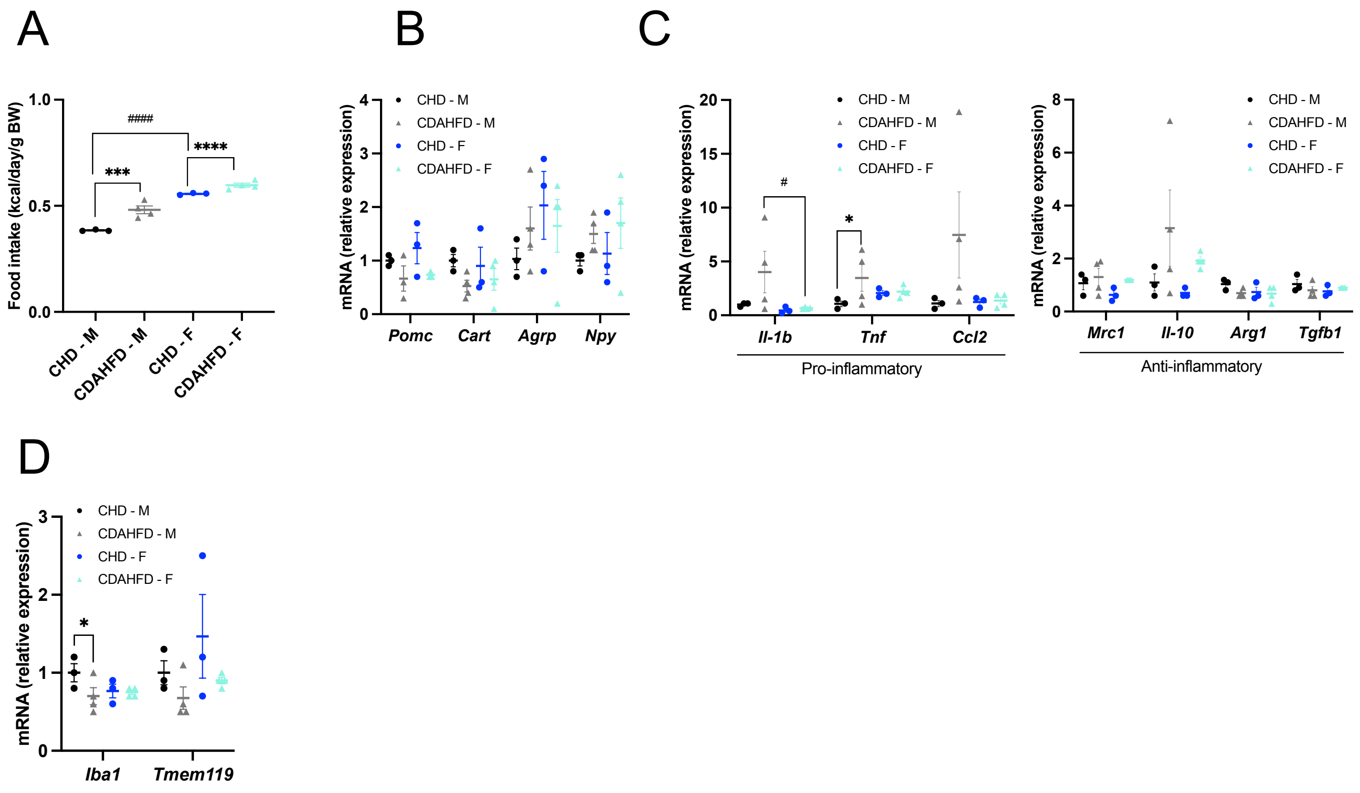

Supplementary Figure 3. Central responses to long-term CDAHFD feeding. Following 14 weeks of chow diet (CHD) or choline-deficient L-amino acid-defined high-fat diet (CDAHFD) feeding, hypothalamic samples were collected from male (M) and female (F) mice for subsequent analyses. Average food intake normalized by body weight (**A**). RT-qPCR analysis of hypothalamic appetite-regulating neuropeptides (**B**), markers of inflammation (**C**) and microglial homeostasis (**D**). Results are presented as mean ± SEM. n= 3 CHD; 3-4 CDAHFD per sex. *p<0.05; **p<0.01; ***p<0.001; ****p<0.0001 (two-way ANOVA for all graphs). Differences between sexes are indicated by * and between diets by #.

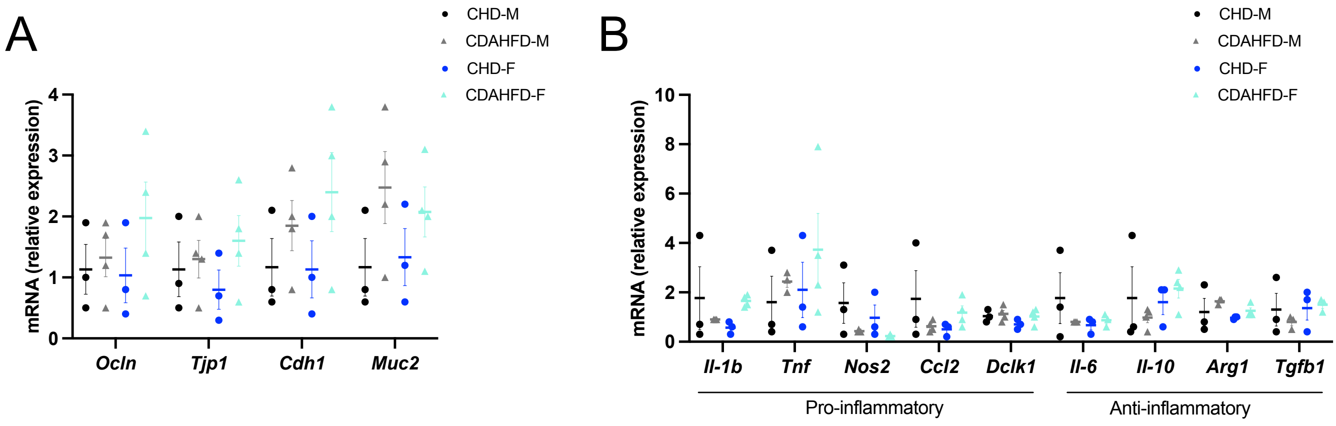

Supplementary Figure 4. Influence of sex in the adaptive response to CDAHFD in the colon. After 14 weeks of chow diet (CHD) or choline-deficient L-amino acid-defined high-fat diet (CDAHFD) feeding, proximal colon was collected from male (M) and female (F) mice for further analysis. RT-qPCR analysis of intestinal barrier-related (**A**) and inflammatory markers expression (**B**). Numeric results are presented as mean ± SEM (n= 3 CHD; 3-4 CDAHFD per sex.); two-way ANOVA for all graphs.
